# A bizarre Early Cretaceous enantiornithine bird with unique crural feathers and an ornithuromorph plough-shaped pygostyle

**DOI:** 10.1038/ncomms14141

**Published:** 2017-01-31

**Authors:** Min Wang, Jingmai K O'Connor, Yanhong Pan, Zhonghe Zhou

**Affiliations:** 1 Key Laboratory of Vertebrate Evolution and Human Origins of Chinese Academy of Sciences, Institute of Vertebrate Paleontology and Paleoanthropology, Chinese Academy of Sciences, Beijing 100044, China; 2 Key Laboratory of Economic Stratigraphy and Palaeogeography of Chinese Academy of Sciences, Nanjing Institute of Geology and Palaeontology, Chinese Academy of Sciences, Nanjing 210008, China

## Abstract

Enantiornithes are the most successful clade of Mesozoic birds. Here, we describe a new enantiornithine bird, *Cruralispennia multidonta* gen. et sp. nov., from the *Protopteryx-*horizon of the Early Cretaceous Huajiying Formation of China. Despite being among the oldest known enantiornithines, *Cruralispennia* displays derived morphologies that are unexpected at such an early stage in the evolution of this clade. A plough-shaped pygostyle, like that of the Ornithuromorpha, evolved convergently in the *Cruralispennia* lineage, highlighting the homoplastic nature of early avian evolution. The extremely slender coracoid morphology was previously unknown among Early Cretaceous enantiornithines but is common in Late Cretaceous taxa, indicating that by 131 million years ago this clade had already experienced considerable morphological differentiation. *Cruralispennia* preserves unusual crural feathers that are proximally wire-like with filamentous distal tips, a new morphotype previously unknown among fossil or modern feathers, further increasing the known diversity of primitive feather morphologies.

## Abstract (web summary)

Although now extinct, Enantiornithes was the most diverse group of birds in the Mesozoic. Here, Wang and colleagues describe a new species of enantiornithine bird from 131 million years ago with features that suggest extensive diversification had occurred in the enantiornithines by this time.

The Enantiornithes are the most diverse group of Mesozoic birds, commonly resolved as the sister group to the Ornithuromorpha, the clade within which modern birds are nested^1^,^2^. Until now, the earliest record of the Enantiornithes is from the Lower Cretaceous Huajiying Formation of northeastern China^3^, the second oldest bird-bearing deposit surpassed only by the German Upper Jurassic Solnhofen Limestones that produces *Archaeopteryx*^3^,^4^,^5^. All vertebrate fossils are from the 130.7 Myr *Protopteryx-*horizon, which records the earliest stage of the well-known Jehol biota^6^,^7^. Hitherto, four birds have been described from this horizon: the basal pygostylian *Eoconfuciusornis*^8^, the most basal enantiornithines *Protopteryx* and *Eopengornis*^3^,^4^ and the ornithuromorph *Archaeornithura*^2^.

Here, we report a new enantiornithine bird from this horizon, *Cruralispennia multidonta* gen. et sp. nov., based on a semi-articulated skeleton preserving feathers. Phylogenetic analysis identifies *Cruralispennia* as more derived than contemporaneous enantiornithines and even some younger taxa, strongly suggesting an even earlier origin for the enantiornithine lineage than previously hypothesized. *Cruralispennia* also displays a number of unusual features, including a plough-shaped pygostyle previously considered unique to the Ornithuromorpha and previously unknown crural feathers. We compare these features with other known basal birds and discuss their functional and evolutionary significance.

## Results

### Systematic palaeontology

         Aves Linnaeus 1758

       Ornithothoraces Chiappe 1995

        Enantiornithes Walker 1981

       *Cruralispennia multidonta* gen. et sp. nov.

 **Etymology**. The generic name is derived from Latin ‘*Cruralis*' and ‘*penna*', referring to the unique feathers on the tibiotarsus; the specific name is derived from Latin ‘*mult*' and ‘*donta*', referring to the numerous dentary teeth.

**Figure 1:**
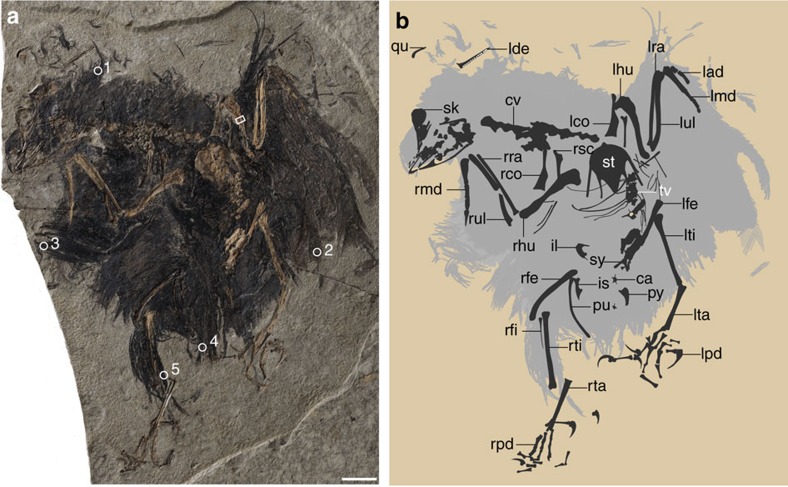
*Cruralispennia multidonta* holotype (IVPP V21711). (**a**) Photograph; (**b**) line drawing. ca, caudal vertebra; cv, cervical vertebra; il, ilium; is, ischium; lad, left alular digit; lco, left coracoid; lde, left dentary; lfe, left femur; lhu, left humerus; lmd, left major digit; lpd; left pedal digits; lra, left radius; lta, left tarsometatarsus; lti, left tibiotarsus; lul, left ulna; pu, pubis; py, pygostyle; qu, quadrate; rco, right coracoid; rfe, right femur; rhu, right humerus; rmd, right major digit; rpd, right pedal digits; rra, right radius; rsc, right scapula; rta, right tarsometatarsus; rti, right tibiotarsus; rul, right ulna; sk, skull; st, sternum; sy, synsacrum; tv, thoracic vertebra. The white circles (numbered 1–5) and box indicate the locations of the feather and histological samples, respectively. Scale bar, 10 mm.

 **Holotype**. IVPP 21711 (housed at the Institute of Vertebrate Paleontology and Paleoanthropology), a nearly fully articulated partial skeleton with associated feathers preserved on a single slab (Fig. 1; Supplementary Table 1).

 **Locality and horizon**. The new specimen is collected from the *Protopteryx-*horizon of the Huajiying Formation at the Sichakou Basin, Fengning County, Hebei Province, northeastern China. Four other birds are reported from the same horizon, *Eoconfuciusornis*, *Protopteryx*, *Eopengornis* and *Archaeornithura*^2^,^3^,^4^,^8^. Stratigraphic correlation and isotopic dating place this horizon at 130.7 Myr ago, late Early Cretaceous^5^,^7^,^9^.

 **Diagnosis**. A small enantiornithine with the following unique features: 14 dentary teeth; abbreviated, plough-shaped pygostyle with a pygostyle/tarsometatarsus length ratio of about 0.28; coracoid mediolaterally narrow with the sternal margin measuring only one-quarter of the proximo-distal length; sternum bearing a V-shaped caudal margin and two pairs of subequal caudal trabeculae; manus shorter than the humerus; postacetabular process of the ilium short and strongly ventrally directed; dorsal process of the ischium more distally placed; and pubis without a distal expansion.

### Description

**Figure 2:**
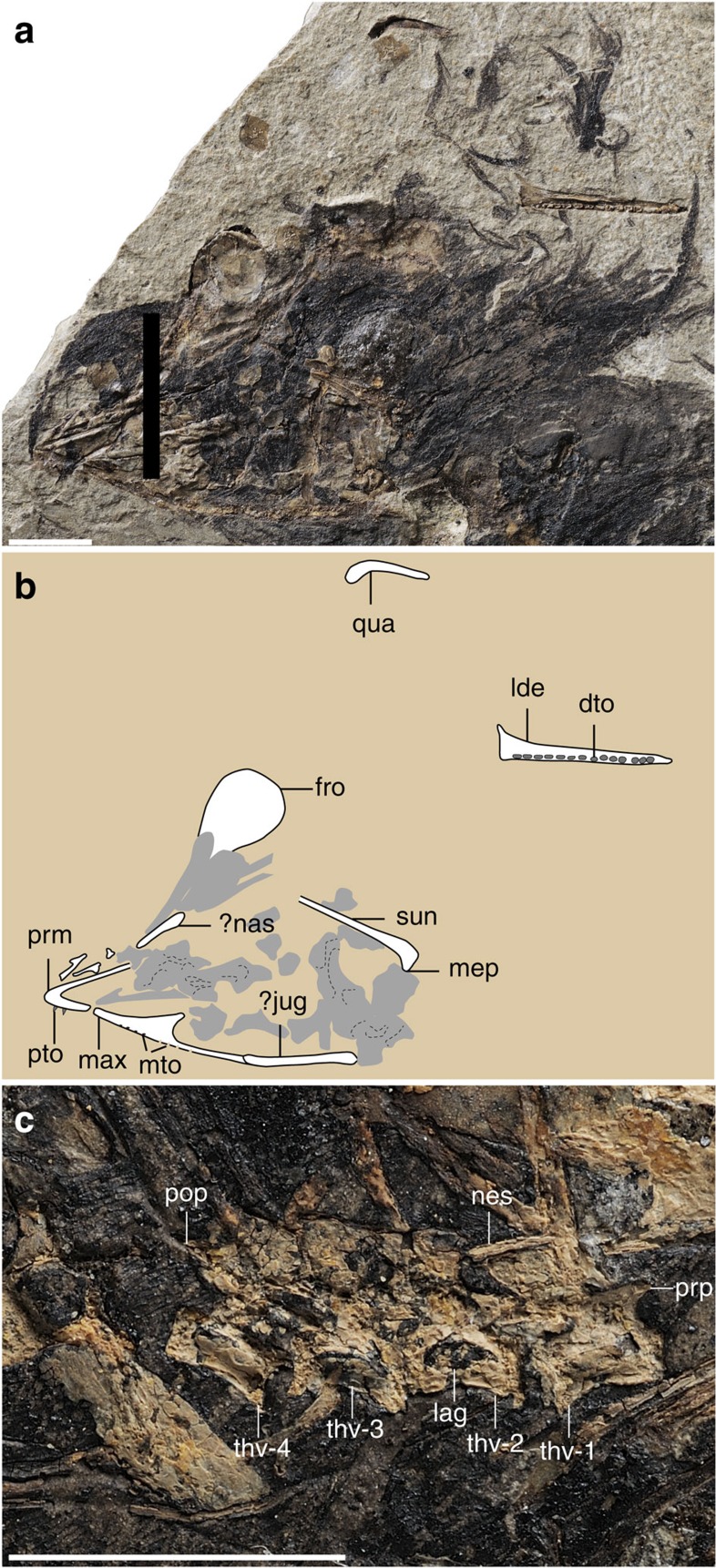
Skull and thoracic vertebrae of *C. multidonta* (IVPP V21711). (**a**) Photograph, (**b**) and line drawing of the skull, (**c**) thoracic vertebrae; dto, dentary tooth; fro, frontal; jug, jugal; lag, lateral groove of centrum; lde, left dentary; max, maxilla; mep, medial process of surangular; mto, maxillary tooth; nas, nasal; nes, neural spine; pop, postzygapophysis; prm, premaxilla; prp, prezygapophysis; pto, premaxillary tooth; qua, quadrate; sun, surangular; thv1–4, preserved thoracic vertebra 1–4. Scale bars, 5mm.

The skull is poorly preserved and partially disarticulated with only a few elements that are clearly identifiable (Fig. 2a,b). The short premaxillary corpus defines a 43° angle with the frontal process, which is considerably larger than in most other enantiornithines, for example, *Protopteryx* (28°), *Parabohaiornis* (26°) and *Eoenantiornis* (30°). Only two premaxillary teeth are visible, but poor preservation is likely obscuring the presence of additional teeth. Typically, enantiornithines have four premaxillary teeth^10^. The maxilla is triradiate in lateral view with a caudodorsally projecting dorsal process. Unlike in *Pengornis*^11^, the dorsal process is imperforate. The maxilla preserves traces of four maxillary teeth. The left frontal is exposed in ventral view; the caudal half is vaulted dorsally. A crescent-shaped element, displaced away from the cranial bones, probably represents the quadrate. It is identical to the laterally exposed left quadrate preserved in the holotype of *Pengornis houi*. As in most enantiornithines, a mandibular symphysis is absent. The left dentary is preserved in dorsal view (Fig. 2a). Fourteen dentary were present, more than in other known enantiornithine (for example, six to ten in bohaiornithids, two in *Protopteryx*^4^, six in *Vescornis*, three in *Longipteryx*) but similar to pengornithids (thirteen in *Pengornis*; *Eopengornis* was estimated to have 12–14 dentary teeth^3^). As in other basal birds with the exception of *Archaeopteryx*^12^, interdental plates are absent. All the dentary teeth are broken, missing their crowns to some extent. The outline of the base of the dentary teeth exhibits considerable variation, becoming progressively more buccolingually compressed caudal in the toothrow, with the width/length ratio declining from 0.85 to 0.43. In the fifth, sixth, tenth and twelfth through fourteenth teeth, the crowns are completely lost, revealing a large tooth pulp (an internal space housing the connective tissue and odontoblasts) occupying nearly the entire cross section of the tooth.

**Figure 3:**
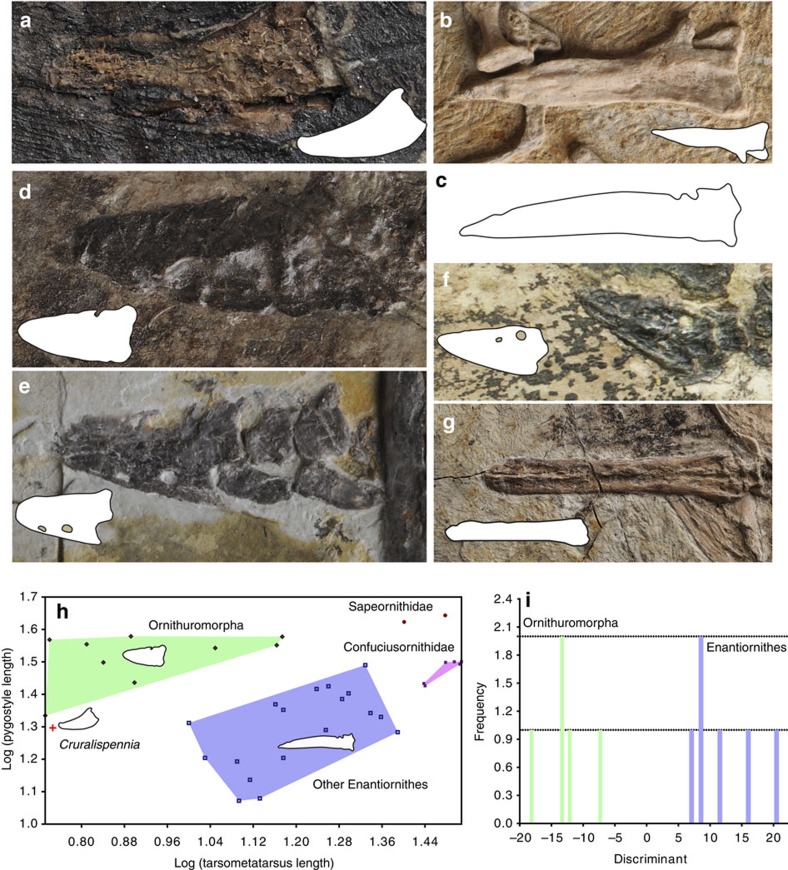
Pygostyle of *C. multidonta* in comparison with other basal birds. (**a**–**e**) photographs and line drawings of the pygostyle in lateral view: (**a**) *Cruralispennia*; (**b**) enantiornithine *Pterygornis*; (**c**) enantiornithine *Concornis*; (**d**) ornithuromorph *Yixianornis*; (**e**) ornithuromorph *Bellulornis*; (**f**) ornithuromorph *Piscivoravis*; (**g**) basal pygostylian *Confuciusornis*. (**h**) Pygostyle length (y-axis) plotted against tarsometatarsal length (*x* axis) to compare the relative length of the pygostyle between groups of Mesozoic birds, showing that the highly abbreviated pygostyle of *Cruralispennia* is distinct from other enantiornithines but comparable to that of the Ornithuromorpha. (**i**) Results of discriminant analysis as histogram showing the Enantiornithes and basal ornithuromorphs plotted along the axis that maximizes their pygostyle differences; the obtained discriminant function suggest that the pygostyle of *Cruralispennia* falls within the morphospace of the Ornithuromorpha.

The vertebral column is incomplete (Fig. 1). Four thoracic vertebrae remain in articulation (Fig. 2c). Although the centra are largely broken, impressions of their lateral surfaces are preserved, indicating that the lateral surface was excavated by a groove in life, a feature characteristic of the Enantiornithes^13^. Only two free caudal vertebrae are preserved (Fig. 1). The pygostyle is fully fused (Fig. 3a). Unexpectedly, the bone is more similar to that of ornithuromorphs than to the elongated form of other enantiornithines (Fig. 3b–f). The pygostyle is abbreviated, having a pygostyle/tarsometatarsus length ratio of 0.28. Similar pygostyle ratios are otherwise known only in ornithuromorphs among Early Cretaceous birds (for example, 0.29 in *Yixianornis*, 0.22 in *Iteravis*, 0.25 in *Archaeorhynchus*; Supplementary Table 2; Fig. 3h). In contrast, the pygostyle is more than half the length of the tarsometatarsus in most enantiornithines, for example, *Vescornis* (0.67), *Protopteryx* (0.69), *Pterygornis* (0.72) and *Sulcavis* (0.79), and in some cases even subequal to or longer than the latter element (for example, *Parabohaiornis* and the Longipterygidae^14^,^15^). Although the Pengornithidae is characterized by a proportionately short pygostyle^3^,^16^,^17^, pygostyle to tarsometatarsus ratios exceed 0.4 in all the known specimens. In more basal pygostylians (Fig. 3g), the same ratio is ∼0.68 in the Sapeornithidae, and in the Confuciusornithidae the robust pygostyle is subequal to or longer than the tarsometatarsus (Fig. 3h; Supplementary Table 2). In *Cruralispennia*, the pygostyle is broad at its proximal end, its dorsoventral height decreasing sharply distally forming a blunt, dorsally upturned distal margin (Fig. 3a). The dorsal margin of the bone is concave, and the ventral margin is convex. All these features are characteristic of the plough-shaped pygostyle of ornithuromorphs in lateral view (Fig. 3d–f)^18^. Multiple lines of evidence suggest that the dorsal curvature of the pygostyle is not the result of deformation: first, the basal avian pygostyle is a fairly robust and stout element, unlikely to be deformed, and no similar deformation has been reported in the pygostyle of specimens of *Protopteryx* and *Eopengornis* from the same locality or any other Jehol enantiornithine; second, the dorsal and ventral margins of the pygostyle are smooth and unbroken (Fig. 3a); and third, slender elements such as the delicate fibula and the pubis, which are more vulnerable to postmortem crushing than the pygostyle, show no sign of deformation, further reinforcing our interpretation that the plough-shaped morphology of the pygostyle is a genuine feature of this new taxon. In contrast, in most enantiornithines this element is more rod-like in lateral view with nearly straight dorsal and ventral margins (Fig. 3b,c); the dorsoventral height remains even along the proximal half of the element, and gently decreases towards the distal end, marking the end of the ventrolateral processes. Sometimes this transition is abrupt forming a distinct distal constriction of the pygostyle^19^, which is notably absent in *Cruralispennia*. The distinct, dorsally upturned distal end of the pygostyle in *Cruralispennia* and many ornithuromorphs is absent in all other known enantiornithines.

**Figure 4:**
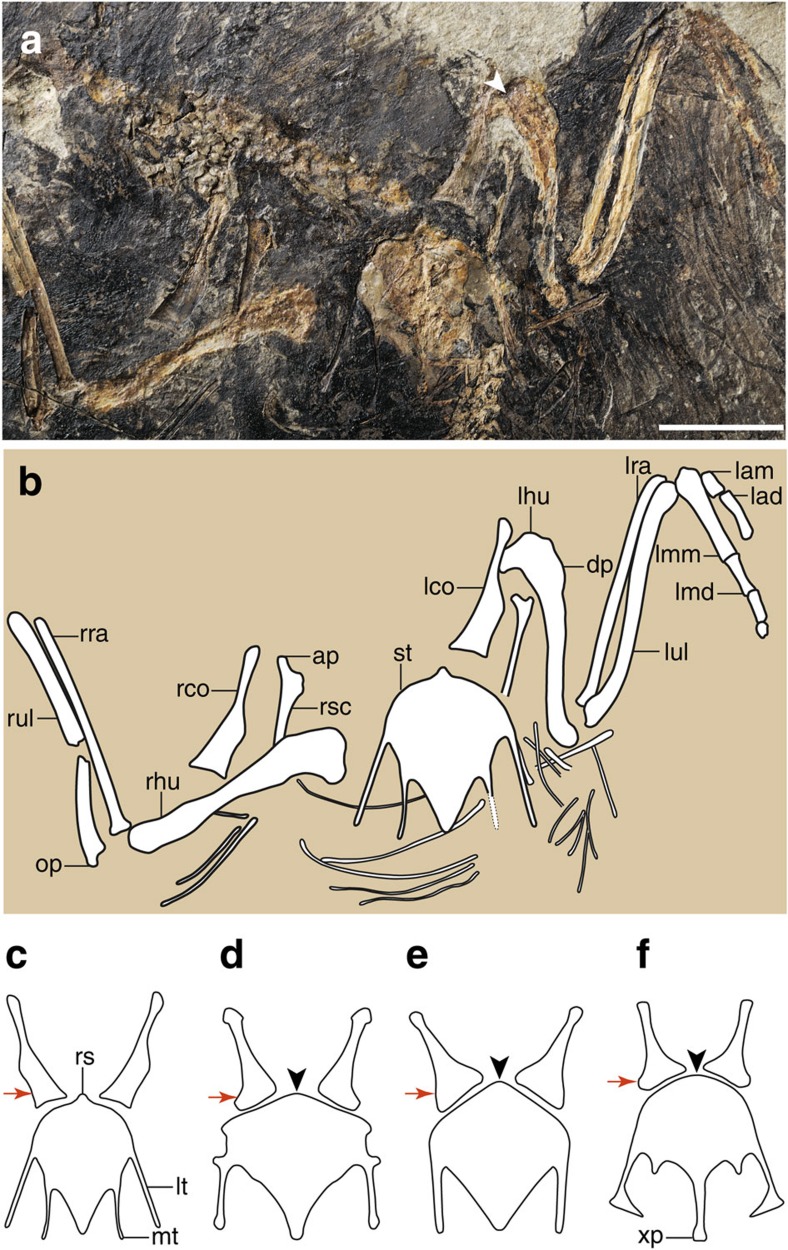
Shoulder and forelimb of *C. multidonta* and some enantiornithines. (**a**) Photograph, (**b**) and line drawing of *Cruralispennia*, IVPP V21711; (**c**–**f**) reconstruction (not to scale) of the coracoids and sternum in *Cruralispennia* (**c**), *Protopteryx* (**d**), *Eopengornis* (**e**) and *Parabohaiornis* (**f**). ap, acromion process; dp, deltopectoral crest; lad, left alular digit; lam, left alular metacarpal; lco, left coracoid; lhu, left humerus; lmd, left major digit; lmm, left major metacarpal; lra, left radius; lt, lateral trabecula; lul, left ulna; mt, intermediate trabecula; op, olecranon process; rco, right coracoid; rhu, right humerus; rra, right radius; rs, rostral spine; rsc, right scapula; rul, right ulna; st, sternum; xp, xiphoid process. The white arrowhead in **a** indicates the circular fossa on the proximocranial surface of the humerus. The lateral margin of the distal half of the coracoid in *Cruralispennia* is weakly concave (red arrow in **c**), in contrast to the strong convex form in other enantiornithines (red arrows in **d**–**f**). The sternum of *Cruralispennia* bears a rostral spine, a structure not present in most enantiornithines (black arrowheads in **d**–**f**). Scale bar, 10 mm.

The strut-like coracoid is considerably more slender than in other Early Cretaceous enantiornithines and basal birds (Fig. 4). The ratio of the sternal margin width to the proximo-caudal length is ∼0.26, significantly smaller than in other Jehol enantiornithines, for example, *Protopteryx* (0.45), *Eopengornis* (0.59), *Eoenantiornis* (0.56) and *Pterygornis* (0.52), with the exception of *Vescornis*. Notably, comparably narrow coracoids are common in Late Cretaceous enantiornithines such as *Enantiornis* and *Neuquenornis*^20^. As in all other enantiornithines with the exception of *Protopteryx*, neither a procoracoid nor a lateral process is developed. The proximal one-third of the coracoid is rod-like, after which the element rapidly expands mediolaterally up to the midpoint of the shaft. Along the sternal half of the coracoid the corpus has a nearly even mediolateral width although the medial and lateral margins are weakly concave (Fig. 4a–c). In comparison, in other enantiornithines the sternal corpus is typically fan-shaped, increasing in width until the sternal margin (Fig. 4d–f)^13^,^20^. The impression left by the coracoid indicates that the sternal half of the coracoid was excavated by a dorsal fossa, a feature widely distributed among enantiornithines^13^,^21^. In Late Cretaceous taxa with comparably narrow coracoids the dorsal excavation is much deeper than in Early Cretaceous enantiornithines including *Cruralispennia*^20^. The scapular acromion process is straight and projects proximodorsally (Fig. 4b), as in other enantiornithines except pengornithids^11^,^17^. The midpoint of the cranial margin of the sternum bears a small cranially projecting process (Fig. 4b,c), a structure probably homologous to the rostral spine of living birds^22^, and also known in the Enantiornithes^23^, but not widely present (absent in *Protopteryx*, *Eopengornis*, the Longipterygidae and the Bohaiornithidae; Fig. 4d,e). The caudal margin of the sternum bears two pairs of subequal trabeculae (Fig. 4c). The lateral trabeculae are caudolaterally oriented. As in *Protopteryx* and pengornithids, these lateral trabeculae lack a distinct distal expansion like that present in most other taxa^15^,^16^. The intermediate trabeculae are slightly more delicate and extend to nearly the same level as the lateral trabeculae, a feature unknown in the Enantiornithes but characteristic of the Ornithuromorpha. The condition in *Cruralispennia* is most reminiscent of the morphology in basal-most ornithuromorph *Archaeorhynchus* in which the subequal processes are elongate and strap-like (short in more derived taxa)^24^. In other enantiornithines the intermediate trabeculae are short and triangular, except *Protopteryx*, in which these processes are only faintly visible^4^, and pengornithids, in which they are absent^3^. Distally, the xiphial region of the sternum is V-shaped, as in the basal enantiornithines *Protopteryx* and pengornithids (Fig. 4c–e). In contrast, it narrows, forming a distinct xiphoid process in other enantiornithines (Fig. 4f)^15^,^17^. The caudal margin of the xiphial region defines an angle of 34°, which is more acute than observed in *Protopteryx* (41°) and pengornithids (40°–75°). As in pengornithids^3^,^17^, the xiphial region and the lateral trabecula terminate at the same level, but the former extends farther caudally in *Protopteryx* (Fig. 4c–e).

The forelimb is short relative to the hindlimb, with an intermembral index (humerus+ulna/femur+tibiotarsus) of 0.97. In contrast, the forelimb is longer in most other enantiornithines (for example, 1.10 in *Protopteryx*, 1.29 in *Eopengornis*, 1.42 in *Pengornis*, 1.15 in *Parabohaiornis*). The robust humerus is shorter than the ulna. The humeral head is concave cranially and its proximal margin is concave centrally, and a circular fossa is developed on the midline of the proximocranial surface (Fig. 4a), features characteristic of the Enantiornithes^13^. The small deltopectoral crest is narrow, less than half of the midshaft width, and extends only along the proximal quarter of the humerus. The ulna is robust, bowed proximally, and has a well-developed blunt olecranon process (Fig. 4a,b). As in more derived birds, the hand (carpometacarpus+major digit) is shorter than the humerus, whereas the hand is longer in contemporaneous basal enantiornithines *Protopteryx* and *Eopengornis*, and more basal birds (for example, *Archaeopteryx*, *Sapeornis* and *Confuciusornis*^3^,^4^,^25^,^26^). As in pengornithids and most other enantiornithines^25^, the alular digit is reduced so that its proximal phalanx ends proximal to the distal end of the major metacarpal (Fig. 4a,b), whereas the two elements terminate at the same level in *Protopteryx*^4^.

The pelvic bones (ilium, ischium and pubis) are poorly preserved. The postacetabular process of the ilium is short and strongly ventrally directed, distinguishable from other enantiornithines (see Supplementary Note 1 for detailed description; Supplementary Fig. 1). The tibiotarsus measures ∼116% of the femoral length. Like most other enantiornithines^15^, the proximal end of the fibula is triangular and the shaft rapidly tapers so that distally the bone is thin and delicate, terminating near the midpoint of the tibiotarsus (Fig. 1; Supplementary Fig. 2). In basal enantiornithines *Protopteryx* and the Pengornithidae, the fibula is unreduced and nearly reaches the lateral condyle of the tibiotarsus^3^,^16^. No free distal tarsals are recognized, and metatarsals II–IV appear to be fused only proximally (Supplementary Fig. 2). The tarsometatarsus is gracile and elongate, measuring 85% of the tibiotarsus (typically closer to half of the tibiotarsus length in most enantiornithines). Metatarsal III is clearly the longest. The trochlea of metatarsal I appears to have articulated above the level of the other metatarsal trochlea. The pedal digits are disarticulated. The non-ungual phalanges are gracile with deep collateral ligamental fovea. The claws are recurved with well-developed flexor processes and lateral surfaces deeply excavated by a deep neurovascular sulcus (Supplementary Fig. 2).

**Figure 5:**
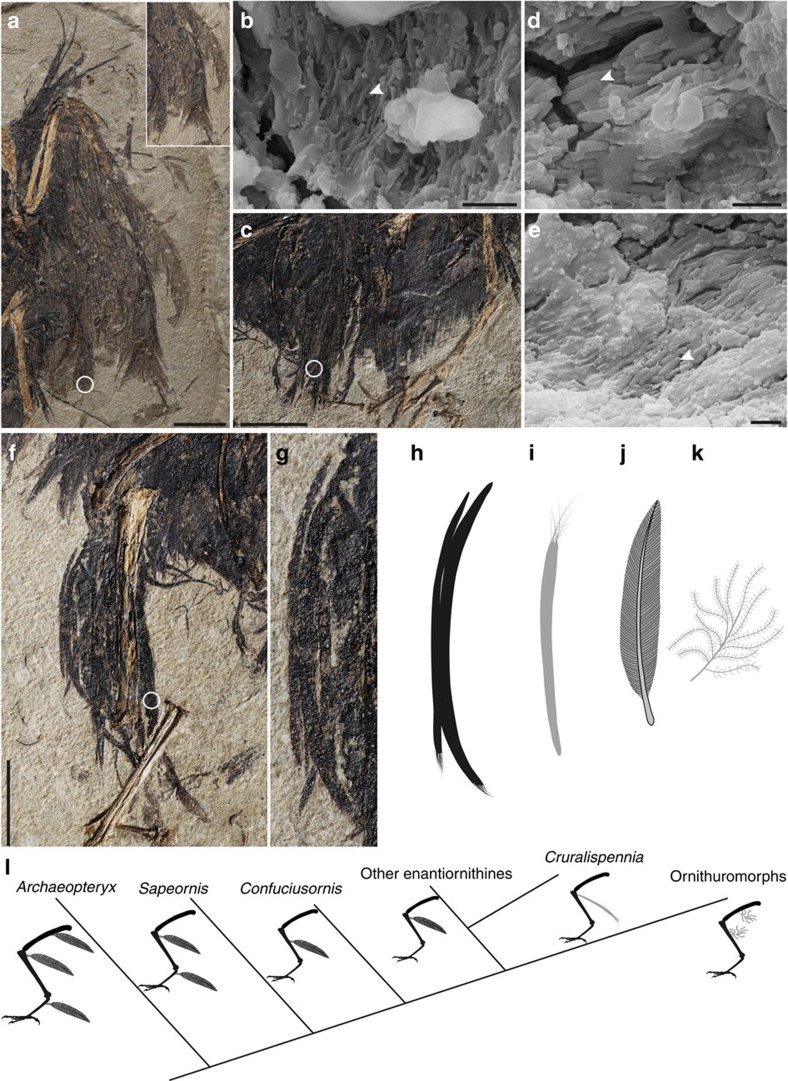
Plumage of *C. multidonta* and leg feather morphotype of basal birds (IVPP V21711). (**a**) Left wing of *Cruralispennia* with inset showing the distal ends of remiges under higher magnification; (**b**) SEM image of wing samples (location indicated by the circle in **a**) showing the melanosomes (white arrowhead); (**c**) tail feathers of *Cruralispennia*; (**d**) SEM image of tail feather samples (circle in **c**); (**f**) right tibiotarsus feathers with two disassociated feathers (**g**); (**e**) SEM image of crural feather sample (circle in **f**); (**h**–**k**) reconstructed the known leg feathers in basal birds: (**h**,**i**) proximally wire-like with filamentous distal tips (PWFDTs) crural feather of *Cruralispennia*; (**j**) model of a pennaceous leg feather; (**k**) modern of a down-like leg feather; (**l**) distribution of leg feather morphotypes among basal birds based on information from literature^34^ and the present study (note that feather size is exaggerated). (**a**,**c**,**f**) Scale bars, 10 mm, and (**b**,**d**,**e**) Scale bars, 2 μm.

The entire skeleton is surrounded by the carbonized remains of feathers with the exception of the rostrum and feet (Fig. 1). The neck feathers are longer along the dorsal margin than the ventral margin. The body feathers appear to be hair-like and rachis-less, as in most other Jehol birds^27^,^28^. The partially folded left wing preserves several bilaterally asymmetrical remiges (Fig. 5a), although the overlap between feathers prevents an accurate assessment of their number. As preserved the feathers are shorter than in other enantiornithines, measuring only twice the length of the manus. The most striking integumentary feature is the crural feathers, best preserved on the right tibiotarsus (Fig. 5f,g). Feathers on the femur are obscured by overlap with feathers from other parts of the body. As in other enantiornithines, feathers are absent on the tarsometatarsus. An array of at least 14 feathers is preserved along the entire length of the right tibiotarsus. These feathers are heavily overlapped but two dissociated feathers clearly preserve the morphology of their proximal and distal ends (Fig. 5g). The two feathers measure 12.1 and 15.8 mm in length, with a subequal width of ∼0.25 mm. They are preserved curved and tapered at the proximal end. The feathers are narrow and wire-like almost the entire length, only distally fraying into individual hair-like barbs that account for <10% the length of the feather (Fig. 5h). The distal hair-like barbs run parallel to each other, similar to other rachis-less body feathers. Whereas in pennaceous feathers the barbs project off a central shaft, and the two vanes form a herringbone structure. Individual barbs cannot be identified in the proximally wire-like portion of any of the fourteen preserved crural feathers, suggesting that the proximal wire-like portion is likely the result of the fusion of individual barbs forming a rachis-like structure. The rachis differs from that in other fossil feathers^27^,^28^,^29^ in that the wire-like portion is proportionally longer than in the down-like body feathers and substantially narrower than in basal birds with an elongated rachis (for example, pennaceous feathers and other morphotypes; 0.37–0.67 mm in *Archaeopteryx*^30^, 1.06 mm in *Confuciusornis*^31^; 2.02 mm in *Eopengornis*). Furthermore, in some feathers from the Jehol biota and especially those from the same locality of *Cruralispennia*, the rachis appears dark in colour often bounded by light vanes laterally^28^. In the crural feathers preserved in *Cruralispennia* the rachises are preserved a uniform dark colour. This new feather morphology, proximally wire-like part with a short filamentous distal tip (PWFDTs), differs from all modern feathers and has not been previously observed in non-avian dinosaurs or basal birds (Fig. 5h–k)^27^,^28^,^29^,^32^. Approximately ten PWFDT-like feathers are preserved projecting from the cranial margin of the left wing (Fig. 5a), suggesting that this feather type is not restricted to the hindlimb. Although these feathers share some features with the proximally ribbon-like pennaceous tail feathers preserved in a juvenile oviraptorosaur *Similicaudipteryx*, individual barbs are readily identifiable along the distal third of the rectrices in the latter and they form pennaceous vanes^29^. Experimental studies have begun to uncover the molecular pathways responsible for the diversity of modern feather morphologies^32^,^33^. Bone morphogenetic protein 4 (*BMP4*) promotes barb fusion and rachis formation, whereas sonic hedgehog (*Shh*) induces apoptosis of the marginal plate epithelia to form individual barbs^33^. The wire-like portion in PWFDTs is likely the result of the overexpression of *BMP4* and/or suppression of *Shh*, notably the same pathways regarded as being responsible for the formation of rachis-dominated rectrices^28^.

Crural feathers are commonly present in both basal and modern birds. In *Archaeopteryx*, *Sapeornis*, *Confuciusornis* and some enantiornithines, the crural feathers are pennaceous with symmetrical vanes (Fig. 5l)^15^,^30^,^34^,^35^. In some enantiornithines and in *Yanornis*, the only Early Cretaceous ornithuromorph preserving leg feathers, the crural feathers are short and have a downy appearance similar to other body feathers (Fig. 5k)^34^, distinct from PWFDTs. In living birds with well-developed crural feathers, such as raptors, the feathers are pennaceous and may offer aerodynamic benefits as air brakes, allowing raptors to achieve a steeper descent when attacking prey^36^. In some other birds such as owls, the feathers have a downy morphology and even extend onto the pedal digits, serving as protection against prey and or thermal insulation^37^. In the absence of pennaceous vanes it is unlikely that the PWFDTs in *Cruralispennia* would have provided much aerodynamic utility. Unfortunately, it is notoriously difficult to ascertain feather function in fossil birds, and possible functions of PWFDTs in *Cruralispennia* include, but are not limited to, insulation, heat shielding, and social signalling. In fact, elongated feathers tend to be proximally narrow, which reduces drag, making it possible to be ornamental but designed to mitigate aerodynamic cost (possible function for PWFDT), similar to the ‘racket-plumes' in the tails of some birds^38^.

Five feather samples, from the skull, right and left wings, tail, and tibiotarsus (Fig. 1), were analysed using scanning electron microscopy (SEM). These feather samples consist of densely packed aligned three-dimensional sub-micrometric bodies (Fig. 5b,d,e; Supplementary Fig. 3), which are interpreted as fossilized melanosomes in following with recent studies^39^,^40^. These microstructures are mostly identified as eumelanosomes based on their elongate, rod shape (0.91–2.61 μm long; 136–473 nm wide)^39^. The proportions of the eumelanosomes vary between sampled regions: the eumelanosomes of the tail are the largest (1.92–2.61 μm long; 380–512 nm wide; Fig. 5e); the eumelanosomes of the skull have the largest aspect ratio (long axis to short axis) averaging 7.14, followed closely by the eumelanosomes in the wings and tail (Supplementary Fig. 3); whereas the crural feathers show the smallest aspect ratio averaging 3.86 (Fig. 5e). Melanosome geometry and feather colour are highly correlated^40^,^41^, and thus the crural feathers most likely represent a different colour from other body parts. This may suggest that these unusual feathers were used in display.

**Figure 6:**
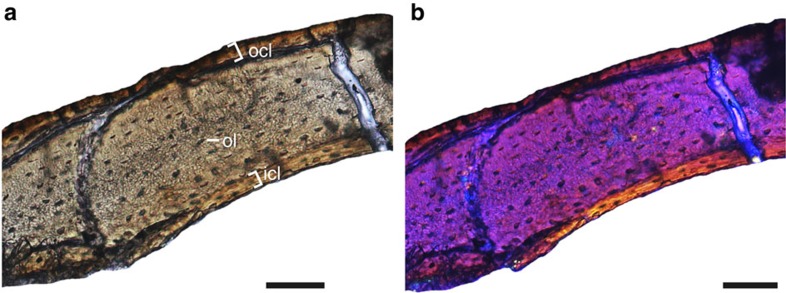
Bone histology of *C. multidonta* holotype (IVPP V21711). (**a**) Transverse section of the right humerus under normal light, and (**b**) polarized light with λ-compensator (see Supplementary Fig. 4 for complete thin section). Abbreviations: icl, inner circumferential layer; ocl, outer circumferential layer; ol, osteocyte lacunae. Scale bars, 50 μm.

The bone tissue of the humerus was sampled to investigate the age of IVPP V21711 and form of growth that characterizes *Cruralispennia*. The transverse section of the humerus is avascular, mainly composed of a thick layer of parallel-fibered bone tissue bounded internally by an inner circumferential layer (ICL)^42^ and externally by an outer circumferential layer (OCL)^42^ (Fig. 6; Supplementary Fig. 4). The medullary cavity is lined by the ICL, which consists of avascular lamellar bone tissue of endosteal origin. The ICL is thin, about one-sixth of the total width of the cortex. A few flattened osteocyte lacunae can be observed. In the middle parallel-fibered layer, the osteocyte lacunae become progressively flatter and more highly parallel organized towards the periosteum. The OCL consists of lamellar bone with a small number of flattened osteocyte lacunae. Growth marks such as lines of arrested growth (LAGs) or annuli are absent. The presence of an ICL and OCL together is widely regarded to indicate that active bone deposition has ceased^42^,^43^, suggesting that growth was complete or nearly complete in IVPP V21711 at the time of its death. Previously osteohistological studies indicate that enantiornithines took several years to reach adult size after which they continued to grow very slowly, evident from the preservation of LAGs in the thick middle layer of more rapidly formed bone and closely spaced rest lines in the slowly formed OCL^42^,^44^. The absence of rest lines in the OCL suggests that IVPP V21711 was a subadult at the time of death. The absence of LAGs in the middle layer suggests that unlike other enantiornithines but similar to *Confuciusornis* and derived ornithuromorphs^45^,^46^, *Cruralispennia* reached near adult size within a year. This suggests that derived growth strategies evolved very early in the Enantiornithes, although the persistence of slower growing lineages even into the Late Cretaceous, reveals that the Enantiornithes were diverse in their developmental strategies^42^,^44^. However, compared with other fast-growing avian lineages (woven or fibrolamellar bone tissue), the bone tissue in *Cruralispennia* is parallel-fibered and avascular, indicating that the bone tissue still formed more slowly than in *Confuciusornis* or ornithuromorphs^45^,^46^.

## Discussion

**Figure 7:**
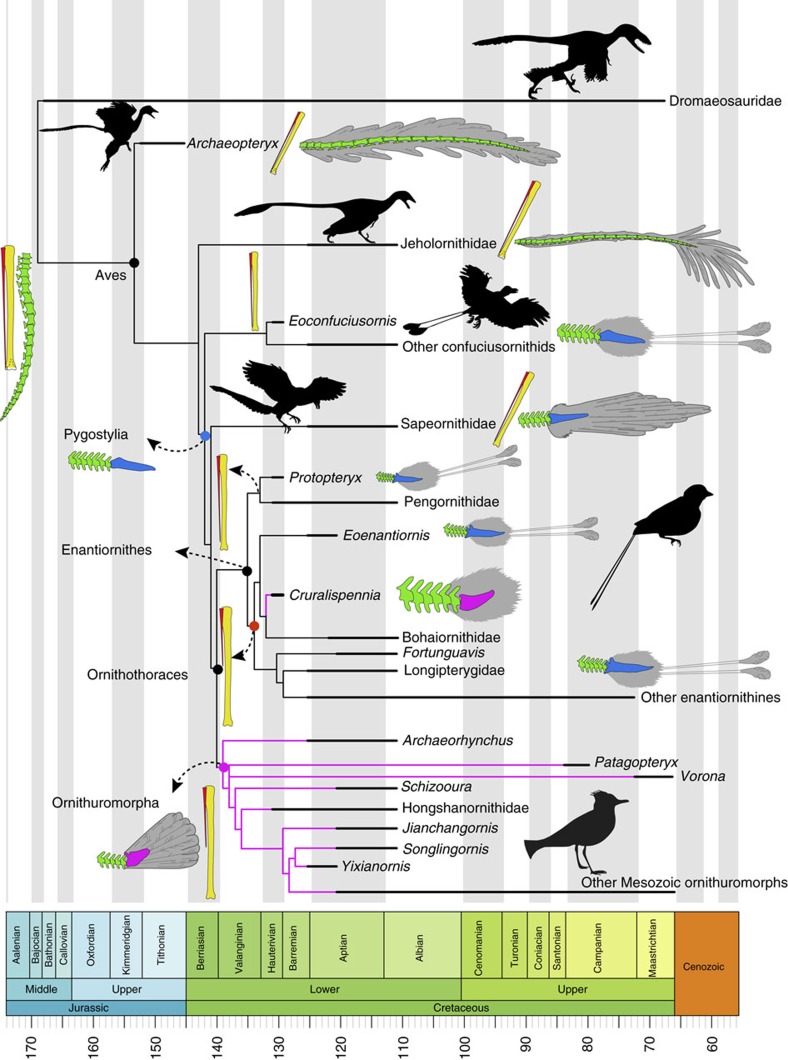
Major transitions of fibula and tail morphology across Mesozoic birds. The tree, simplified from the strict consensus tree produced by our phylogenetic analysis, is time-scaled using the ‘minimal branch length' method with a minimum branch length of 1 Myr (see Supplementary Fig. 5 for complete result and node support values). The thick lines indicate the temporal range of fossil taxa. An elongated fibula (in red) is developed in non-ornithothoracine Aves, and the most basal enantiornithines *Protopteryx* and Pengornithidae; a reduced fibula is convergently evolved in the Ornithuromorpha and derived enantiornithines. *Cruralispennia* preserves a plough-shaped pygostyle (in pink), which has long been considered unique to Ornithuromorpha (the pink branches). The apparent absence of tail fanning in *Cruralispennia* indicates that the plough-shaped pygostyle and tail fanning is evolutionarily decoupled in this lineage (the silhouettes were from ref. 47; the reconstruction of tail feathers is on basis of refs 3, 30).

The new specimen IVPP V21711 is referrable to the Enantiornithes based on the presence of the following enantiornithine features: the humeral head is concave cranially and its proximal margin is concave centrally; a circular fossa is developed on the midline of the proximocranial surface of the humerus; the strut-like coracoid lacks a procoracoid process; and the scapula bears a long acromion process^13^. The new specimen can be readily identified as a new species, *C. multidonta* gen. et sp. nov., based on the presence of several features otherwise unknown in the Enantiornithes, including subequal lateral and medial sternal trabeculae and a plough-shaped pygostyle. Our comprehensive phylogenetic analyses place *Cruralispennia* in a relatively derived position within the Enantiornithes (Fig. 7; Supplementary Fig. 5). Surprisingly, despite being among the oldest enantiornithines, *Cruralispennia* is more derived than contemporaneous taxa *Protopteryx* and *Eopengornis*, as well *Eoenantiornis—*a taxon five million years younger, whereas other contemporaneous enantiornithines are always resolved as basal most taxa. However, despite the presence of a number of derived features (for example, narrow coracoid, plough-shaped pygostyle), *Cruralispennia* falls outside the node that includes most enantiornithines strongly suggesting these features are autapomorphies of the *Cruralispennia* lineage. The stratigraphic-phylogenetic discrepancy incurred by the discovery of *Cruralispennia* changes how we view the temporal span of early bird evolution. To estimate how this new taxon affects origination estimates, the phylogeny was stratigraphically calibrated. The result indicates that divergences between basal avian lineages occurred earlier than previously considered, pushing the enantiornithine-ornithuromorphs split back one million years from previous estimates (Fig. 7)^47^.

*Cruralispennia* shows several morphological features that are derived within the Enantiornithes, and one feature that is more characteristic of the Ornithuromorpha. The hand is shorter than the humerus, whereas a long hand is plesiomorphically retained by *Protopteryx*, *Eopengornis*, and more basal birds such as *Archaeopteryx*, *Confuciusornis* and *Jeholornis*. The fibula is reduced, as preserved measuring less than half the length of the tibiotarsus (Supplementary Fig. 2). However, this bone is nearly as long as the tibia in non-avian theropods^48^, basal birds (for example, *Archaeopteryx* and *Sapeornis*), and contemporaneous enantiornithines *Protopteryx* and *Eopengornis* (Fig. 7)^3^,^4^,^8^. The adaptive value of a short fibula in birds has long being a standing point of uncertainty, but is presumed to be a byproduct of selection favoring a longer tibia^49^. One developmental study shows that the loss of the distal fibular epiphysis (functioning as the growth plate) during embryonic development causes the reduction of the fibula^50^, resulting in the greater fibula-tibial disparity seen in modern birds. The presence of a long fibula persists in basal avian lineages present in the younger Yixian and Jiufotang formations, including the basal enantiornithine lineage, the Pengornithidae^26^. Most basal ornithuromorphs have a short fibula, including *Archaeornithura* from the Huajiying Formation^2^,^18^,^24^. The relatively long fibula present in the basal ornithuromorph *Patagopteryx* from the Late Cretaceous of Argentina still fails to contact the proximal tarsals^51^. Although the large range of fibula lengths in basal and modern ornithuromorphs prevents generalization, the available fossils indicate that a short fibula is widely distributed in the basal ornithuromorphs, although this may be somewhat exaggerated by poor preservation of the splint-like distal fibula in small Early Cretaceous taxa. However, a long fibula is clearly present in the basal enantiornithines *Protopteryx* and the Pengornithidae, the latter family including taxa from both the Huajiying and Jiufotang formations^3^,^4^,^17^. This element is strongly reduced in all the other more derived enantiornithines^15^. *Cruralispennia* documents the oldest record of fibular reduction in the Enantiornithes. The available fossil evidence indicates that postmorphogenetic changes related to fibula development evolved independently in the Ornithuromorpha and derived lineages of the Enantiornithes (Fig. 7).

During the early avian evolution the tail underwent a profound transformation^52^,^53^. *Archaeopteryx* and *Jeholornis* inherited a plesiomorphically long bony tail from their dinosaurian ancestors, which was replaced by a pygostyle in more derived birds (Fig. 7). Unfortunately, little is known about this dramatic transition, and it is unclear when this feature evolved. Furthermore, the pygostyle of living birds is very different from the morphology in primitive birds, thus the origin of the pygostyle and the origin of the ‘modern pygostyle' are separate issues. The pygostyle of the enantiornithine *Cruralispennia* appears similar to that of ornithuromorphs and living birds (Fig. 3a,d–f)^22^, contributing new information to this issue through the addition of unexpected homoplasy. As in ornithuromorphs, the pygostyle in *Cruralispennia* is strongly abbreviated. Although in the basal pygostylians clades—the Sapeornithidae and Confuciusornithidae, and most enantiornithines the length of the pygostyle exceeds the combined length of the free caudals (Fig. 7)^26^, in *Cruralispennia* we estimate the pygostyle was shorter than the free caudal series as in the Ornithuromorpha. To quantify the relative brevity of the pygostyle between Mesozoic birds, tarsometatarsus and pygostyle lengths were obtained and plotted against each other to construct a two-dimensional morphospace. The results show that *Cruralispennia* is closer to the ornithuromorph morphospace than to that of the Enantiornithes, Confuciusornithidae and Sapeornithidae (Fig. 3h). Although detailed features are obscured by compression and two-dimensional preservation, the general shape of the pygostyle of *Cruralispennia* is indistinguishable from that of ornithuromorphs, particularly with regards to the upturned distal end (Fig. 3a,d–f). In contrast, this bone is straight in other enantiornithines (Fig. 3b,c), the Confuciusornithidae (Fig. 3g), and Sapeornithidae. A discriminant analysis was performed, using three linear measurements—lengths of the tarsometatarsus and pygostyle, and the dorsoventral height of the pygostyle at its proximal end—to explore whether the pygostyle of *Cruralispennia* can be statistically assigned to the morphospace of the Enantiornithes or the Ornithuromorpha. The Enantiornithes and Ornithuromorpha are well separated by these variables (Fig. 3i), and the resultant discriminant function indicates that the pygostyle of *Cruralispennia* falls within the ornithuromorph morphospace (Supplementary Table 3). In living birds, the pygostyle is surrounded by paired soft tissue structures, the rectricial bulbs. The rectricial bulbs and their musculature allow birds to control the shape of the tail to match flight conditions, making the tail an important component of the flight apparatus^54^. Previously, the co-occurrence of the plough-shaped pygostyle with a fan-shaped array of rectrices only in the Ornithuromorpha led some to argue that these two structures coevolved with the rectricial bulbs^2^,^17^,^18^,^53^,^55^. A fan-tail was assumed to be present in the longipterygid enantiornithine *Shanweiniao*^56^, but the feathers are poorly preserved as faint, incomplete traces, severely limiting interpretations; re-examination of the specimen has suggested that that rectrices were not aerodynamic (not forming a fan), but were elongate and rachis-dominated, a feather morphology widely distributed among the Enantiornithes^17^. Recently, O'Connor *et al*.^17^ described a pengornithid *Chiappeavis*, the first enantiornithine to unequivocally preserve a fan-shaped array of rectrices. The pygostyle of *Chiappeavis* and other pengornithids is proportionally shorter than in other enantiornithines, but still longer than in ornithuromorphs. Notably, other pengornithids preserving rectrices preserve a pair of elongate rachis-dominated feathers similar to those found in typical enantiornithine taxa with a long robust pygostyle. In most enantiornithines the tail consists of normal body feathers and a pair of elongate rectrices that are inferred to be present in males only and lack the ability to fan (Fig. 7)^28^. Given the morphological similarity between the pygostyle of *Cruralispennia* and that of the Ornithuromorpha, and observed correlations between pygostyle morphology and rectricial morphology in Aves, we would predict that *Cruralispennia* would have a fan-shaped array of rectrices. However, no elongated tail feathers are preserved in IVPP V21711, and the tail consists of normal, non-pennaceous body feathers (Fig. 5), suggesting that a rectricial bulb is absent. This lends support to some studies that suggest pygostyle morphology is not as strongly linked to the evolution of the rectricial bulb and tail fanning as previously claimed^17^,^57^. It appears that a plough-shaped pygostyle evolved in parallel in the Ornithuromorpha and in at least one enantiornithine lineage, for example, *Cruralispennia* (Fig. 7), but in the latter this morphology does not appear related to associated soft tissue and musculature. The function of the enantiornithine plough-shaped pygostyle is unknown at this time. The discovery of this morphology in the Enantiornithes contributes to the tally of numerous instances of homoplasy that characterize early avian evolution^1^.

The Early Cretaceous Jehol biota with its numerous exceptionally preserved fossil birds has greatly contributed to our understanding of early bird history^1^,^6^. A majority of Jehol birds are from the Yixian and Jiufotang formations, which generally exhibit an increase in biodiversity through time^1^,^6^,^18^,^20^. As the earliest phase of the Jehol radiation, the Huajiying Formation has the potential to shed light on the diversification of many important groups, including Aves. Taxonomic diversity is considerably lower in the Huajiying Formation compared with the overlying Yixian and Jiufotang formations^1^,^58^. Only four avian species from three clades have been previously described from the Huajiying Formation, each representing the basal-most members of their respective clades with the exception of *Archaeornithura*^2^,^3^,^8^, and now *Cruralispennia*. Although in some regards, the Huajiying Formation records the early stages in the diversification of the Jehol avifauna, the numerous derived features preserved in *Cruralispennia* and the ornithuromorph *Archaeornithura* clearly indicate that avian bauplans were already quiet diverse by 130.7 Myr with numerous morphologies already being utilized by several avian lineages. We suggest this reflects high evolutionary rates in the early history of the Enantiornithes and Ornithuromorpha (possibly representing an adaptive radiation). Alternatively, this pattern may reflect incomplete sampling of the early avian record; increased collecting efforts targeting older deposits close to the Jurassic/Cretaceous boundary may prove especially valuable for better understanding about early avian evolution.

## Methods

### Phylogenetic analysis

To investigate the systematic position of IVPP V21711, we performed phylogenetic analyses using a modified version of the Mesozoic bird dataset of Wang *et al*.^2^. The new dataset consists of 262 morphological characters scored for 56 Mesozoic birds, one outgroup (represented by the Dromaeosauridae), and two neornithines *Gallus gallus* and *Anas platyrhynchos* (Supplementary Data 1 and 2). The dataset was analysed using the parsimony method in the TNT software package^59^, with the following settings: all characters were equally weighted; 33 characters were treated as ordered; and memory allowed space for up to 10,000 trees was set. We performed an unconstrained heuristic search starting with Wagner trees first running; 2,000 replicates of random stepwise addition (branch swapping trees: tree-bisection-reconnection, TBR), maintaining 10 trees at each step, and collapsing branches to create polytomies if the minimum branch length was zero. This produced six most parsimonious trees (MPTs) of 1,011 steps (consistency index=0.362; retention index=0.681; Supplementary Data 3). We ran an additional round of TBR to fully explore each of the tree islands. No additional MPTs were recovered. The general placement of important taxa within the six MPTs is summarized in the strict consensus tree. To obtain support index for each node, we calculated both the Bootstrap and Bremer values (Supplementary Fig. 5). Bootstrap values were retained by performing 1,000 replicates in TNT using the default settings. Bremer values were calculated using the bremer script embedded in the TNT programme. The strict consensus tree is nearly resolved with the exception of small polytomies among derived enantiornithines.

### Time-scaled phylogenies

The phylogeny of Mesozoic birds (the strict consensus tree) was time-scaled using the method in Wang and Graeme^47^, to estimate the divergence times of basal avian lineages. The temporal ranges of fossil taxa in question were determined by the lower and upper bounds of the fossil-bearing horizons from which they are from (refs 60, 61); to incorporate the uncertainty in temporal ranges, a randomization analysis were conducted^47^,^61^. When phylogenies involving fossil taxa are time-scaled, branches with zero-length duration may be produced, because the internal node shares the same date as its oldest descendant^62^. The zero-length branches were smoothed using the ‘minimum branch length' (‘mbl') method^63^, which imposes a minimum branch length of 1 Myr. The ‘equal' method^62^, which smooths zero-length branches by allowing them to share duration equally with preceding non-zero-length branches, is not used here because in a previous study it significantly pushed the divergence times back into the late Jurassic, and we consider the ‘mbl' method to be more conservative^47^. All these analyses were performed in R (v. 3.2.3 (ref. 64)) using the timePaleoPhy function in the package paleotree^65^. The addition of IVPP V21711 brings small changes compared with the result in Wang and Graeme^47^ that also used the ‘mbl' method: in the new time-scaled phylogeny, the origination date of the Enantiornithes has been pushed back 1 Myr earlier (Supplementary Fig. 5). The result is not unexpected, because although IVPP V21711 is phylogenetically more derived than *Eoenantiornis*, it is stratigraphically older, coming from the same locality as basal taxa *Protopteryx* and *Eopengornis*. Therefore, the branches subtending to *Protopteryx+*Pengornithidae, and the clade including *Eoenantiornis*, IVPP V21711 and more derived taxa, is pushed back in light of the discovery of *Cruralispennia*.

### Feather samples preparation

Five feather samples are collected from different regions of the body: near the skull, the left and right wings, tail and the right tibiotarsus (corresponding to the number 1–5 in Fig. 1). Samples were mounted on stubs with carbon tape and coated with gold (60 s). The samples were observed and photographed using a Leo1530VP scanning electron microscope with a SE2 detector.

### Histological preparation

The bone thin section was prepared following the standard methods described by Lamm^66^. Because the surfaces the long bones are badly abraded, only the left humerus was sampled from a point as close to the mid-diaphysis as preservation allowed (Fig. 1). Samples were embedded in one-component resin (EXAKT Technovit 7200), and hardened in a light polymerization device (EXAKT 520) for 12 h. Histological thin sections were cut transversely using an accurate circular saw (EXAKT 300CP). Sections were mounted on frosted glass slides with adhesive (EXAKT Technovit 7230), and then grounded down using the EXAKT 400CS grinding system until the desired optical contrast was obtained. The bone sections were observed by light microscopy under normal and polarized lights (Zeiss AX10). Images were captured using a digital camera (Zeiss AxioCam MRc5).

### Pygostyle morphospace analysis

The lengths of the pygostyle and tarsometatarsus were measured to quantify relative pygostyle length in different Mesozoic avian clades. These elements were selected because they are commonly preserved and can be compared between a large number of taxa. Most specimens were measured directly, although data for a few taxa were taken from the literature (Supplementary Table 2). The dataset includes 36 Mesozoic taxa, consisting of the Confuciusornithidae (*n*=6), Sapeornithidae (*n*=2), Enantiornithes (*n*=19), and Ornithuromorpha (*n*=9). The measurements were log-transformed and plotted in a two-dimensional space using the PAST software (v. 2.17c)^67^. Most Mesozoic bird specimens are preserved in two-dimensions (including nearly all Jehol birds), and the pygostyle is most commonly preserved in different views, preventing the use of more sophisticated methods such as geometric morphometrics. Tentatively, we use the proximal dorsoventral height of the pygostyle and pygostyle length to capture how rapidly the height of the pygostyle tapers caudally with respective to the tarsometatarsus length. For a pygostyle of a given length, this ratio (pygostyle height/pygostyle length) is larger in ornithuromorphs than in enantiornithines. To test if differences in pygostyle shape are statistically significant, three measurements were subjected to a discriminant analysis using the PAST software. The discriminant analysis uses predetermined groups (here, Enantiornithes and Ornithuromorpha) to create an axis to maximize the differences between these groups, plotting these groups along the resultant axis using a histogram; a discriminant function is reconstructed, which can be used to classify new specimens (in this case, *Cruralispennia*)^68^. The equality of the means of the groups was tested using the Hotelling's *T*-squared test, a multivariate analogue to the *t* test, and a *P*-value was obtained^68^. The proximal dorsoventral height of the pygostyle could only been accurately measured in six enantiornithines other than *Cruralispennia* and seven ornithuromorphs. All the measurements were log-transformed to normalize the distributions. The discriminant analysis confirms that Enantiornithes and Ornithuromorpha are morphologically distinct on the basis of these measurements (Fig. 3i; *P*<0.01), and no specimen is incorrectly classified (for example, an ornithuromorph is classified to the Enantiornithes and vice versa). By using the resultant discriminant function, the pygostyle of *Cruralispennia* can be grouped to the ornithuromorph morphospace (Supplementary Table 3).

### Nomenclatural act

This published work and the nomenclatural act it contains have been registered in ZooBank, the proposed online registration system for the International Code of Zoological Nomenclature (ICZN). The ZooBank LSID (Life Science Identifier) can be resolved and the associated information viewed through any standard web browser by appending the LSID to the prefix ‘ http://zoobank.org/'. The LSID for this publication is: urn:lsid:zoobank.org:pub:BA1344A0-5FC7-41EC-A228-3D4686DC32A5.

### Data availability

The data are available in the Supplementary Information files and also from the authors by request.

## Additional information

**How to cite this article:** Wang, M. *et al*. A bizarre Early Cretaceous enantiornithine bird with unique crural feathers and an ornithuromorph plough-shaped pygostyle. *Nat. Commun.* **8,** 14141 doi: 10.1038/ncomms14141 (2017).

**Publisher's note:** Springer Nature remains neutral with regard to jurisdictional claims in published maps and institutional affiliations.

## Supplementary Material

Supplementary Information Supplementary Figures, Supplementary Tables, Supplementary Notes and Supplementary References

Supplementary Data 1 Description of morphological character used in the phylogenetic analysis (from Wang et al. 2015)

Supplementary Data 2 Dataset of 262 morphological characters for the 59 taxa included in the phylogenetic analysis.

Supplementary Data 3 The six most parsimonious trees resultant from the phylogenetic analysis.
