# Supplementary material for: A bizarre Early Cretaceous enantiornithine bird with unique crural feathers and an ornithuromorph plough-shaped pygostyle
Source: Nat Commun. 2017 Jan 31;8:14141. doi: 10.1038/ncomms14141 (PMC5290326; doi:10.1038/ncomms14141)
Supplement: Supplementary Information — Supplementary Figures, Supplementary Tables, Supplementary Notes and Supplementary References [file ncomms14141-s1.pdf]

Supplementary Figures

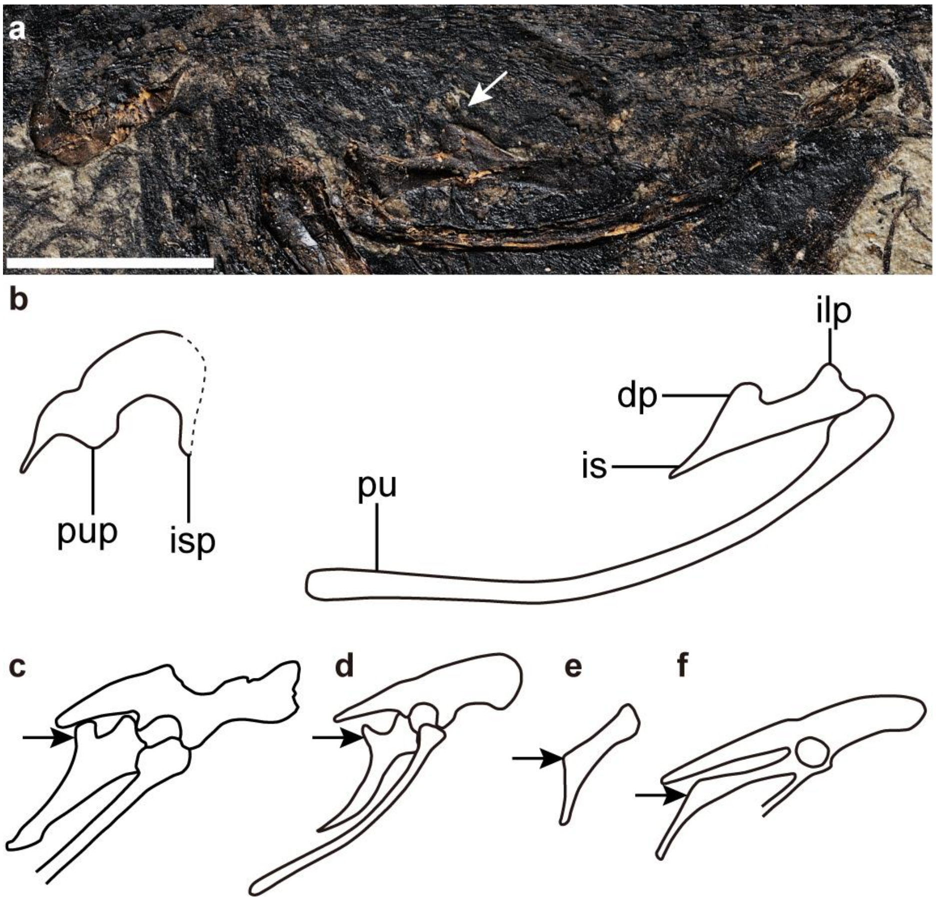

Supplementary Figure 1. Pelvis of *Cruralispennia multidonta* (holotype, IVPP V21711), in comparison with other basal birds. (**a**) Photograph, and (**b**) line drawing of the pelvic bones of *Cruralispennia*; (**c**–**f**) reconstructed pelvis of selected basal birds: enantiornithines *Linyiornis* (**c**) and *Parabohaiornis* (**d**), ornithuromorphs *Yixianornis* (**e**, only ischium) and *Gansus* (**f**). Abbreviations: dp, dorsal process; ilp, iliac peduncle of ischium; is, ischium; isp, ischiadic peduncle of ilium; pu, pubis; pup, pubic peduncle of ilium. The dorsal process is distally located on the dorsal margin of the ischium in *Cruralispennia* (arrow in **a**), recalling the condition in some basal ornithuromorphs (arrows in **e** and **f**); in contrast, that process is more proximally located in enantiornithines (arrows in **c** and **d**). Scale bars in (**a**) equal 5 mm.

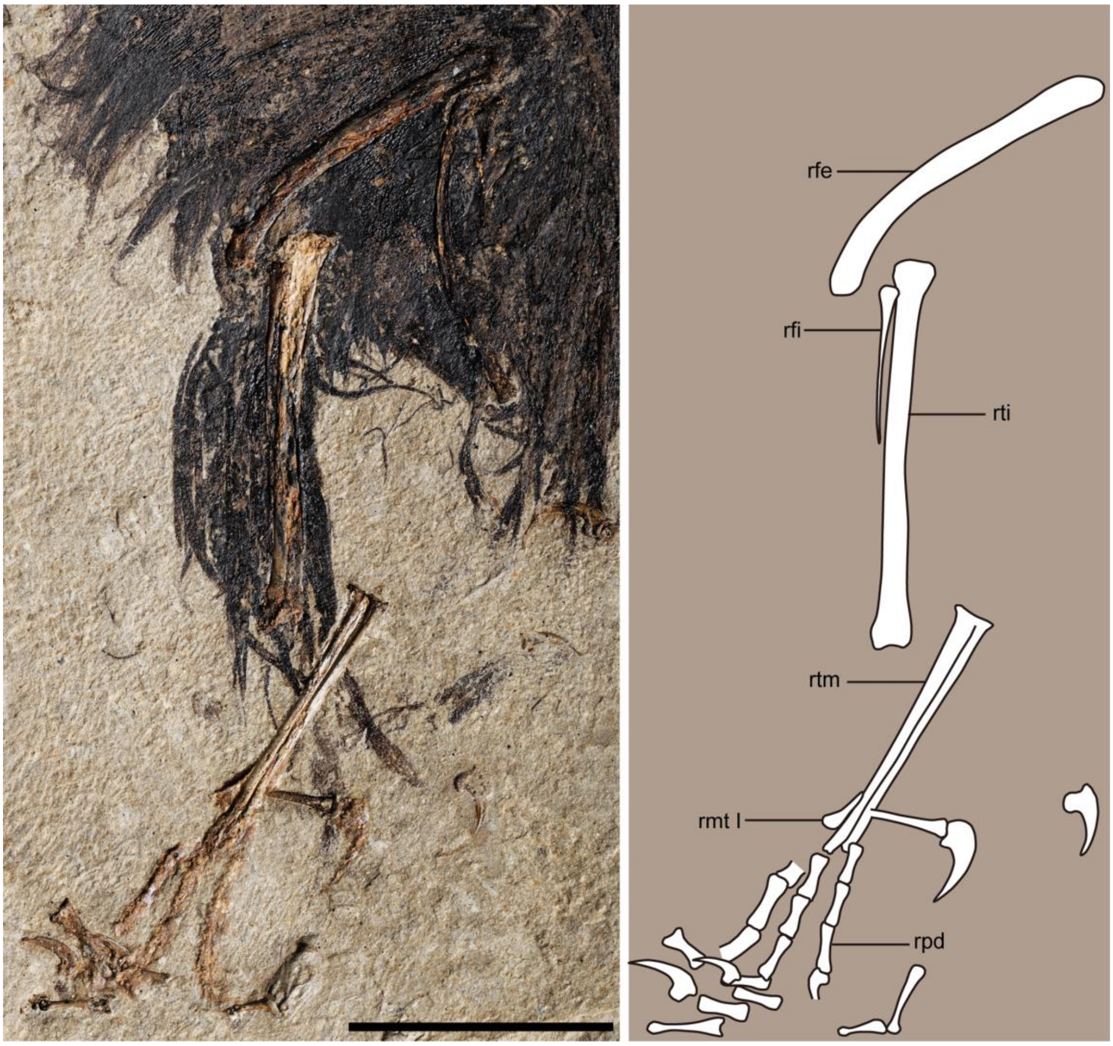

Supplementary Figure 2. Photograph and line drawing of the hindlimb of *Cruralispennia multidonta* holotype (IVPP V21711). Abbreviations: rfe, fight femur; rfi, fight fibula; rmt I, right metatarsal I; rpd, right pedal digits; rti, right tibiotarsus; rtm, right tarsometatarsus. Scale bar, 10 mm.

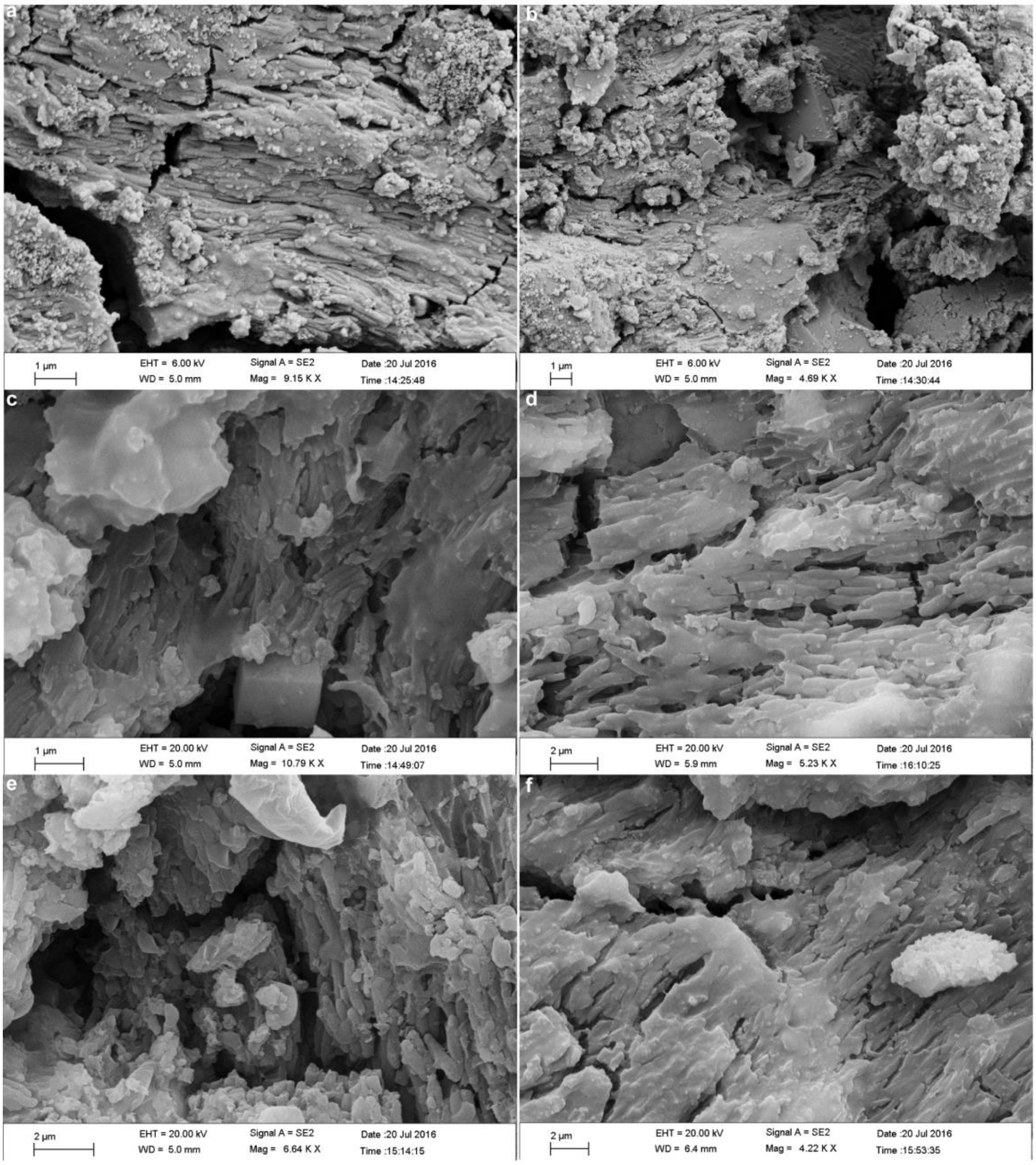

Supplementary Figure 3. Scanning electron microscopy images of the feathers of *Cruralispennia multidonta* (IVPP V21711) showing the fossilized melanosomes. (**a**,**b**) Samples from the skull feathers; (**c**) left wing; (**d**) right wing; (**e**) tail feathers; (**f**) right tibiotarsus feathers. The localities of feather samples are indicated in Figure 1.

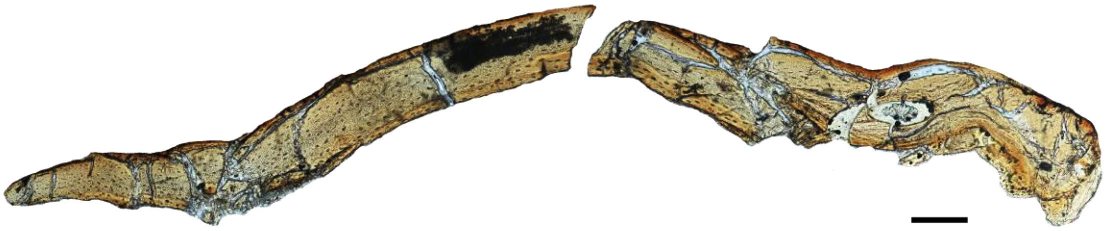

Supplementary Figure 4. Bone histology of the right humerus of *Cruralispennia multidonta* (IVPP V21711) under normal light. Scale bar, 100 μm.

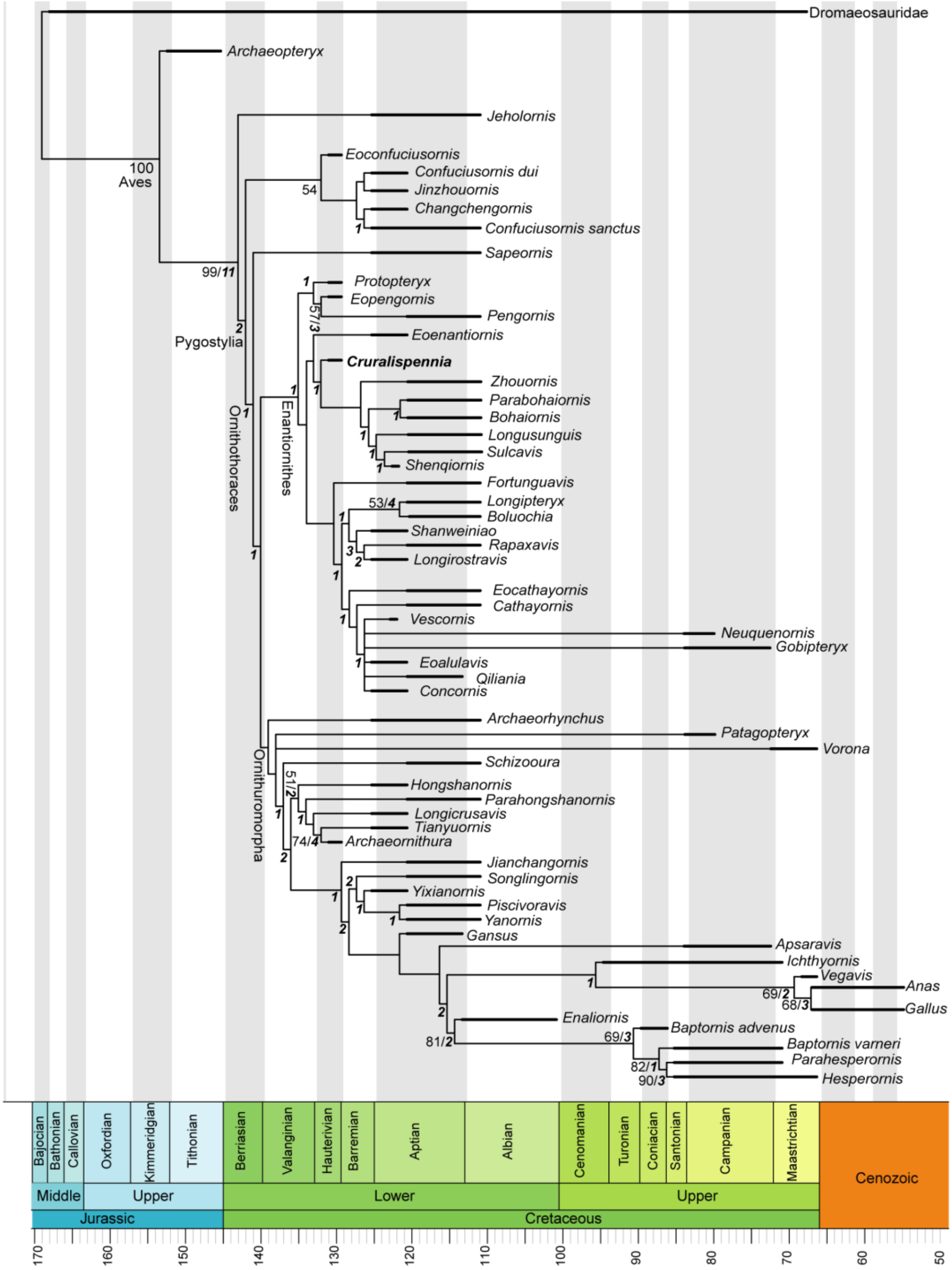

Supplementary Figure 5. Time-scaled phylogeny of Mesozoic birds. The phylogeny is the strict consensus tree of the six most parsimonious trees (tree length = 1011 steps, consistency index = 0.362; retention index = 0.681) derived from the cladistic analyses. The tree is time-scaled using the minimal branch length method with a minimum branch length of 1 Ma. The Bremer and Bootstrap values are indicated in bold italic and normal formats, respectively.

Supplementary Tables

Supplementary Table 1. Selected measurements of *Cruralispennia multidonta* IVPP V21711 (in millimeters)

Element	Measurements
Coracoid length	12.37
Coracoid width at the sternal margin	3.24
Humerus length	19.35
Ulna length	22.48
Radius length	21.35
Carpometacarpus length	8.85
Pygostyle	5.58
Ischium length	6.82
Pubis length	18.12
Femur length	19.75
Tibiotarsus length	23.08
Tarsometatarsus length	19.71

Supplementary Table 2. Selected measurements (in millimeters) of basal birds used in the pygostyle length/tarsometatarsus length plot, and the discriminant analysis. The pygostyle height represents the dorsoventral height at the proximal end of the bone. Most of the data are collected by first hand on the corresponding specimen, with a few from literature.

Taxon	Collection No.	Pygostyle length	Tarsometatarsus length	Pygostyle height
Confuciusornis sanctus	IVPP V13175	30.08	31.51	
Confuciusornis sanctus	IVPP V13172	27.56	26.69	
Confuciusornis sanctus	IVPP V13156	31.23	31.66	
Confuciusornis sanctus	IVPP V13171	31.98	31.13	
Confuciusornis sanctus	IVPP V13168	27.41	27.13	
Confuciusornis sanctus	IVPP V13178	32.21	31.67	
Sapeornis chaoyangensis	IVPP V13276	25.11	42.41	
Sapeornis chaoyangensis	IVPP V22531	30.04	43.93	
Vescornis hebeiensis	130722	10.72	16.00	3.00
Protopteryx fengningensis	IVPP V11665	21.32	30.90	
Pterygornis dapingfangensis	IVPP V20729	12.30	15.60	3.20
Fortunguavis xiaotaizicus	IVPP V18631	19.84	25.30	
Parabohaiornis martini	IVPP V18691	18.00	19.50	
Parabohaiornis martini	IVPP V18690	21.80	22.00	
Longusunguis kurochkini	IVPP V17964	22.80	21.40	
Sulcavis geeorum	BMNHph 000805	19.30	24.30	5.6
Iberomesornis romerali[1]	LH22	13.56	12.00	3.12
Dapingfangornis sentisorhinus	LPM-B0027	15.00	16.00	4.95
Zhouornis hani	CNUVB 0903	17.30	26.10	
Longipteryx chaoyangensis[2]	IVPP V12325	24.50	19.20	
Longirostravis hani[2]	IVPP V11309	13.00	13.70	
Shanweiniao cooperorum[2]	DNHM D1878	12.40	11.80	
Rapaxavis pani[2]	DNHM D2522	14.50	23.40	
Chiappeavis magnapremaxillo[3]	STM29-11	15.00	22.50	5.85
Pengornis houi[3]	IVPP V15336	18.20	26.60	
Parapengornis eurycaudatus[3]	IVPP V18687	10.00	20.50	
Cruralispennia multidonta	IVPP V21711	5.58	19.71	2.11
Yixianornis grabaui	IVPP V12631	7.92	27.30	3.25
Iteravis huchzermeyeri	IVPP V18958	6.93	31.50	4.29
Archaeorhynchus spathula	IVPP V20312	5.40	21.60	3.02
Jianchangornis microdonta[4]	IVPP V16708	5.50	37.00	
Schizooura lii	IVPP V16861	6.45	35.84	

Taxon	Collection No.	Pygostyle length	Tarsometatarsus length	Pygostyle height
Bellulornis rectusunguis	IVPP V17970	11.20	34.90	4.70
Piscivorous lii	IVPP V17078	14.58	35.60	6.99
Dingavis longimaxilla	IVPP V20284	7.80	37.90	
Yanornis martini	IVPP V12558	14.93	37.80	

Supplementary Table 3. Result of discriminant analysis. Three linear measurements—pygostyle length (**pl**), tarsometatarsus length (**tl**), and dorsoventral height of the proximal pygostyle (**ph**)—are used in a discriminant analysis. The pygostyle of Ornithuromorpha and Enantiornithes (*Cruralispennia* not included) are distinctly separated along the axis that maximizes their differences concerning the three measurements (p <0.01; Fig. 3i). Using the resultant discriminant function and subtracting the offset value, the pygostyle of *Cruralispennia* can be classified into the morphotype of Ornithuromorpha (discriminant value < 0).

Hotelling's t^2	68.499
F-value	17.759
p-value	0.001185
Discriminant function	42.859*Log(**pl**)-67.803*Log(**tl**)+7.3551*Log(**ph**)
Offset value	-42.5834

Supplementary Notes

Supplementary Note 1: Additional anatomical description of the pelvis

The pelvic bones (ilium, ischium and pubis) are probably only partially fused at the level of the acetabulum; the right ischium and pubis are preserved in articulation (Supplementary Fig. 1a). A piece of bone preserved craniomedial to the articulated right femur and acetabulum is interpreted as the postacetabular portion of the right ilium in lateral view. If this interpretation is correct, this element preserves a morphology unlike that observed in other enantiornithines. The postacetabular process is sharply tapered and strongly angled ventrally, extending ventrally beyond the level of the ischiadic peduncle (Supplementary Fig. 1b). In contrast, in other enantiornithines the postacetabular process is typically triangular and gently narrows into a blunt distal margin with little to no ventral deflection, e.g., *Sinornis*, *Linyiornis*, *Protopteryx* and bohaiornithids (Supplementary Fig. 1c,d). The post-ischiadic peduncle portion of the postacetabular process is extremely short, only as long as the width of the base of the ischiadic peduncle (Supplementary Fig. 1b). In contrast, the same part is at least twice as long as the latter in other enantiornithines (Supplementary Fig. 1c,d). The ischium is shorter than the pubis, slightly more than half of the length of the pubis. Located just cranial to the midpoint, the ischium bears a distinct, cranially directed triangular process on its dorsal margin (Supplementary Fig. 1b), similar to the condition in some basal ornithuromorphs, e.g., *Yixianornis*, *Gansus* and *Iteravis* (Supplementary Fig. 1e,f)[5], although it is more distally positioned

in *Cruralispennia*. Notably, in other enantiornithines the process is more proximally located and even reaches the ilium to enclose the ilioischiadic foramen (Supplementary Fig. 1c,d). The pubes lack a pubic boot, a feature commonly present in enantiornithines (Supplementary Fig. 1b)[6-8], instead only weakly expanding in dorsoventral height distally.

Supplementary References

1. O'Connor, J. K. A systematic review of Enantiornithes (Aves: Ornithothoraces). (University of Southern California, 2009).

2. O'Connor, J. K., Zhou, Z. & Zhang, F. A reappraisal of *Boluochia zhengi* (Aves: Enantiornithes) and a discussion of intraclade diversity in the Jehol avifauna, China. *J. Syst. Palaeontol.* **9,** 51–63 (2011).

3. O'Connor, J. K., Zheng, X. & Hu, H., Wang, X. & Zhou, Z. The morphology of *Chiappeavis magnapremaxillo* (Pengornithidae: Enantiornithes) and a comparison of aerodynamic function in Early Cretaceous avian tail fans. *Vertebr. Palasiat*. (in press)

4. Zhou, Z., Zhang, F. & Li, Z. A new basal ornithurine bird (*Jianchangornis microdonta* gen. et sp. nov.) from the Lower Cretaceous of China. *Vertebr. Palasiat*. **47,** 299–310 (2009).

5. Clarke, J. A., Zhou, Z. & Zhang, F. Insight into the evolution of avian flight from a new clade of Early Cretaceous ornithurines from China and the morphology of *Yixianornis grabaui*. *J. Anat*. **208,** 287–308 (2006).

6. Zhou, Z., Clarke, J. & Zhang, F. Insight into diversity, body size and morphological evolution from the largest Early Cretaceous enantiornithine bird. *J. Anat*. **212,** 565–577 (2008).

7. O'Connor, J. K. *et al.* An enantiornithine with a fan-shaped tail, and the evolution of the rectricial complex in early birds. *Curr. Biol.* **26,** 114–119 (2016).

8. Wang, M., Zhou, Z., O'Connor, J. K. & Zelenkov, N. V. A new diverse enantiornithine family (Bohaiornithidae fam. nov.) from the Lower Cretaceous of China with information from two new species. *Vertebr. Palasiat*. **52,** 31–76 (2014).
