# Supplementary material for: A bizarre Early Cretaceous enantiornithine bird with unique crural feathers and an ornithuromorph plough-shaped pygostyle
Source: Nat Commun. 2017 Jan 31;8:14141. doi: 10.1038/ncomms14141 (PMC5290326; doi:10.1038/ncomms14141)
Supplement: Supplementary Data 3 — The six most parsimonious trees resultant from the phylogenetic analysis. [file ncomms14141-s4.doc]

**Supplementary Data 3.**

The six most parsimonious trees resultant from the phylogenetic analysis (the tree files are exported using PAUP software package version v.4.0b10 (ref. 1).

#NEXUS

Translate

1 Dromaeosauridae,

2 Archaeopteryx,

3 Jeholornis,

4 Sapeornis,

5 Boluochia,

6 Longipteryx,

7 Longirostravis,

8 Rapaxavis,

9 Shanweiniao,

10 Concornis,

11 Gobipteryx,

12 Neuquenornis,

13 Eoalulavis,

14 Vescornis,

15 Qiliania,

16 Cathayornis,

17 Eocathayornis,

18 Fortunguavis,

19 Eoenantiornis,

20 Shenqiornis,

21 Sulcavis,

22 Longusunguis,

23 Bohaiornis,

24 Parabohaiornis,

25 Zhouornis,

26 Cruralispennia,

27 Pengornis,

28 Eopengornis,

29 Protopteryx,

30 Vorona,

31 Schizooura,

32 Jianchangornis,

33 Songlingornis,

34 Yanornis,

35 Piscivoravis,

36 Yixianornis,

37 Apsaravis,

38 Ichthyornis,

39 Vegavis,

40 Anas,

41 Gallus,

42 Hesperornis,

43 Parahesperornis,

44 Baptornis_varneri,

45 Baptornis_advenus,

46 Enaliornis,

47 Gansus,

48 Hongshanornis,

49 Longicrusavis,

50 Archaeornithura,

51 Tianyuornis,

52 Parahongshanornis,

53 Patagopteryx,

54 Archaeorhynchus,

55 Confuciusornis_sanctus,

56 Changchengornis,

57 Jinzhouornis,

58 Confuciusornis_dui,

59 Eoconfuciusornis

tree'PAUP_1'=[&R](1,(2,(3,((4,(((((((5,6),((7,8),9)),(((((10,(11,12)),13),(14,15)),16),17)),18),(19,(((((20,21),22),(23,24)),25),26))),((27,28),29)),(((30,(31,((32,((33,((34,35),36)),((37,((38,(39,(40,41))),((((42,43),44),45),46))),47))),(48,((49,(50,51)),52))))),53),54))),(((55,56),(57,58)),59)))));

tree'PAUP_2'=[&R](1,(2,(3,((4,(((((((5,6),((7,8),9)),(((((10,(11,12)),13),(14,15)),16),17)),18),(19,(((((20,21),22),(23,24)),25),26))),((27,28),29)),(((30,53),(31,((32,((33,((34,35),36)),((37,((38,(39,(40,41))),((((42,43),44),45),46))),47))),(48,((49,(50,51)),52))))),54))),(((55,56),(57,58)),59)))));

tree'PAUP_3'=[&R](1,(2,(3,((4,(((((((5,6),((7,8),9)),((((((10,15),(11,12)),13),14),16),17)),18),(19,(((((20,21),22),(23,24)),25),26))),((27,28),29)),(((30,(31,((32,((33,((34,35),36)),((37,((38,(39,(40,41))),((((42,43),44),45),46))),47))),(48,((49,(50,51)),52))))),53),54))),(((55,56),(57,58)),59)))));

tree'PAUP_4'=[&R]1,(2,(3,((4,(((((((5,6),((7,8),9)),((((((10,15),(11,12)),13),14),16),17)),18),(19,(((((20,21),22),(23,24)),25),26))),((27,28),29)),(((30,53),(31,((32,((33,((34,35),36)),((37,((38,(39,(40,41))),((((42,43),44),45),46))),47))),(48,((49,(50,51)),52))))),54))),(((55,56),(57,58)),59)))));

tree'PAUP_5'=[&R](1,(2,(3,((4,(((((((5,6),((7,8),9)),((((((10,15),12),13),(11,14)),16),17)),18),(19,(((((20,21),22),(23,24)),25),26))),((27,28),29)),(((30,(31,((32,((33,((34,35),36)),((37,((38,(39,(40,41))),((((42,43),44),45),46))),47))),(48,((49,(50,51)),52))))),53),54))),(((55,56),(57,58)),59)))));

tree'PAUP_6'=[&R](1,(2,(3,((4,(((((((5,6),((7,8),9)),((((((10,15),12),13),(11,14)),16),17)),18),(19,(((((20,21),22),(23,24)),25),26))),((27,28),29)),(((30,53),(31,((32,((33,((34,35),36)),((37,((38,(39,(40,41))),((((42,43),44),45),46))),47))),(48,((49,(50,51)),52))))),54))),(((55,56),(57,58)),59)))));

**Supplementary Reference**

1. Swofford, D. L. *PAUP**. *Phylogenetic Analysis Using Parsimony (*and Other Methods)*. Version 4 (Sinauer Associates, 2002).
